# The Altered Supramolecular Structure of Dopamine D2 Receptors in *Disc1*-deficient Mice

**DOI:** 10.1038/s41598-018-20090-0

**Published:** 2018-01-26

**Authors:** Taichi Onishi, Hirokazu Sakamoto, Shigeyuki Namiki, Kenzo Hirose

**Affiliations:** Department of Neurobiology, Graduate School of medicine, The University of Tokyo, Tokyo, 113-0033 Japan

## Abstract

*Disc1* is a susceptibility gene for psychiatric disorders including schizophrenia. It has been suggested that excess transmission through dopamine type 2 receptors (D2Rs) in the striatum is an underlying mechanism of pathogenesis. In this study, we used super-resolution microscopy to study the distribution of D2Rs at the nanoscale in mice lacking exons 2 and 3 of *Disc1* (*Disc1*-deficient mice). We found that D2Rs in the nucleus accumbens (NAc) of wild-type mice form nanoclusters (~ 20,000 nm^2^), and that *Disc1*-deficient mice have larger and more D2R nanoclusters than wild-type mice. Interestingly, administration of clozapine reduced the size and spatial distribution of the nanoclusters only in *Disc1*-deficient mice. Moreover, we observed that medium spiny neurons in the NAc of *Disc1*-deficient mice had reduced spine density on their dendrites than did wild-type mice, and this was also reversed by clozapine administration. The altered D2R nanoclusters might be morphological representations of the altered dopaminergic transmission in disease states such as schizophrenia.

## Introduction

Excess dopaminergic transmission via dopamine type 2 receptors (D2Rs) in the striatum has been suggested to associate with psychotic symptoms. The association between the dopaminergic hyperactivity and psychosis has been inferred from the positive correlation between the clinical efficacy of antipsychotics and their binding affinity to D2Rs, which is expressed abundantly in the striatum^1^. Furthermore, an experimental result that the expression of D2Rs in the striatum is elevated in schizophrenia patients also supports the association^2^. Although these studies suggest that striatal dopaminergic hyperactivity contributes to the pathogenesis of psychosis, the mechanism which underlies increased dopaminergic transmission remains to be elucidated.

*Disrupted-in-schizophrenia-1* (*Disc1*) is a susceptibility gene for major psychiatric disorders. *Disc1* was initially discovered in a Scottish pedigree with a large number of family members that were diagnosed with psychiatric disorders such as bipolar disorder, major depression and schizophrenia^3^. Mice with *Disc1* mutations exhibit abnormal behaviours in the pre-pulse inhibition test, methamphetamine administration and in the elevated plus maze, consistent with symptoms of schizophrenia^4–6^. *Disc1* mutant mice are reported to have alterations in the dopaminergic system that are strongly associated with enhanced D2R expression. For example, a positron emission tomography and autoradiography study revealed that mice expressing dominant-negative *Disc1* exhibited increased binding of radioligands to D2Rs in the medial striatum^4^. Moreover, mice lacking exons 2 and 3 of *Disc1* (*Disc1*-deficient mice) showed enhanced D2R mRNA in the nucleus accumbens (NAc)^7^.

Although the hypothesis that the spatial distribution of synaptic molecules affects synaptic transmission has been tested from the theoretical view^8^, the hypothesis has not been investigated experimentally because of the limited resolution of the light microscopy. The super-resolution microscopy invented recently enabled to observe detailed molecular distribution with up to ~20 nm resolution^9^. A study utilizing a super-resolution microscopy experimentally revealed that the presynaptic fusion of vesicle containing glutamate preferentially occurs at nanoscale assembly of Rab3-interacting molecules^10^. This study provided an experimental basis of the hypothesis. Because the nanoscale molecular structure which affects the glutamatergic transmission is validated, we predicted that the correlating nanoscale molecular structure with the dopaminergic transmission would also be observed and that the altered nanoscale molecular distribution of D2Rs exist in psychiatric disorders as a morphological representation of the excess dopaminergic transmission in the striatum.

We chose *Disc1*-deficient mice^6^ as our disease model, and used a combination of conventional and super-resolution microscopy. We found that D2Rs constitute nanoscale clusters of approximately 20,000 nm^2^ in size in the NAc of WT mice. The size and density of these D2R clusters were increased in the *Disc1*-deficient mice; this change was reversed to wild-type (WT) levels by the administration of the antipsychotic drug, clozapine. Our data suggest that altered spatial distribution of D2Rs may provide a molecular basis of the hyperdopaminergic activity observed in psychosis.

## Results

### Subregion-specific alteration of D2R expression in the striatum of *Disc1*-deficient mice

**Figure 1:**
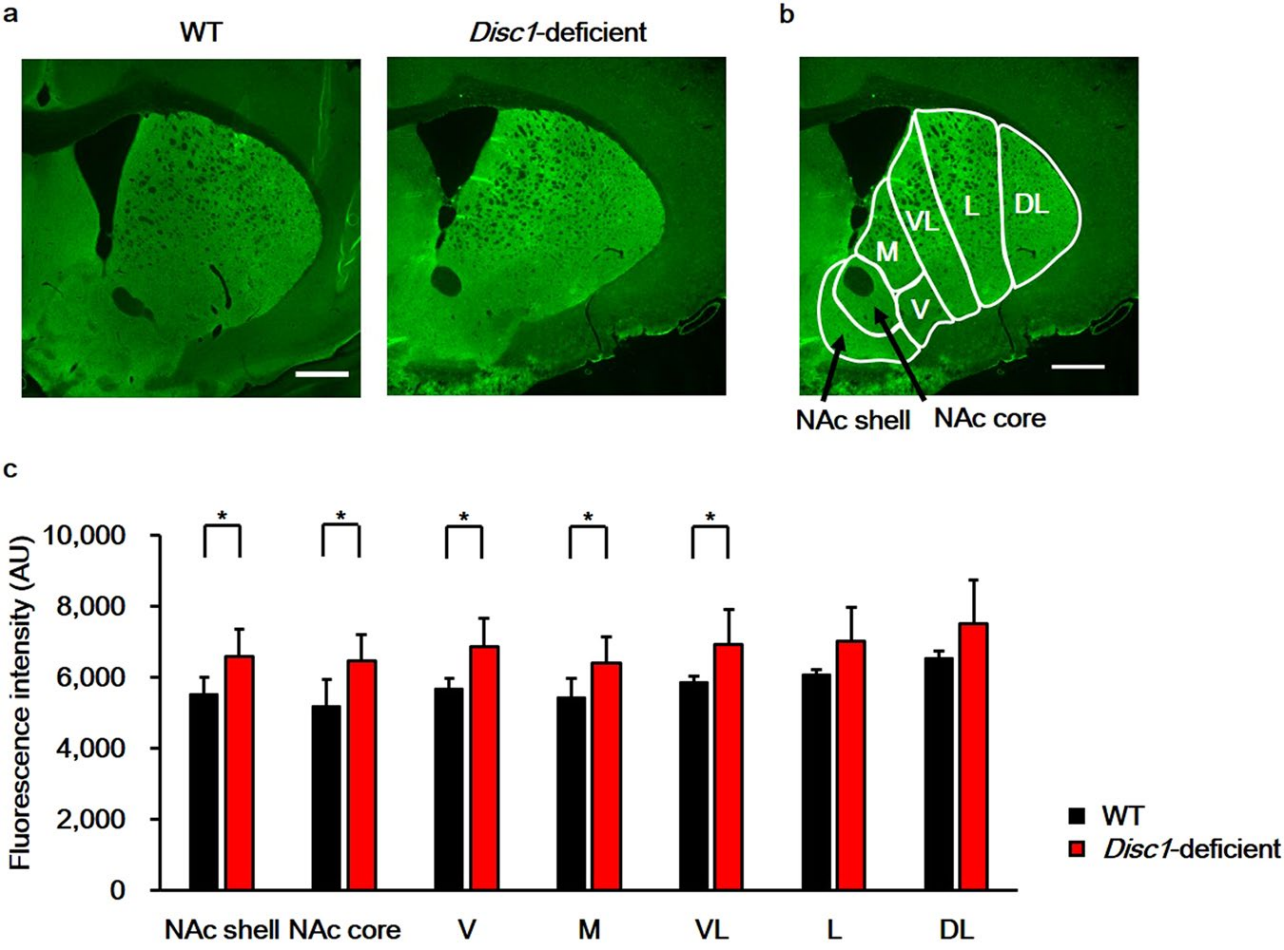
Dopamine type 2 receptor (D2R) expression is increased in the striatal subregions of *Disc1*-deficient mice. (**a**) Representative images of D2R expression in the striatum of wild-type (WT) mice (left) and *Disc1*-deficient mice (right). Scale bar indicates 500 μm. (**b**) Map of the striatum subregions. Scale bar indicates 500 μm. (**c**) Quantification of D2R immunoreactivity in each of the striatal subregions. Eleven images from 6 mice for each genotype were quantified and analysed using the two-tailed Student’s *t*-test (NAc shell: p = 0.019, NAc core: p = 0.015, V: p = 0.013, M: p = 0.027, VL: p = 0.044, L: p = 0.054, DL: p = 0.10). Error bars indicate standard deviation (SD). *p < 0.05, NAc: nucleus accumbens, V: ventral striatum; M: medial striatum; VL: ventrolateral striatum; L: lateral striatum; DL: dorsolateral striatum.

A previous study has shown that D2R mRNA levels are increased in the NAc of *Disc1*-deficient mice^5^. However, whether or not the protein expression level is also altered was not investigated. In a first series of experiments, immunofluorescent staining and conventional imaging of the striatum demonstrated increased D2R immunoreactivity in *Disc1*-deficient mice compared to WT mice (Fig. 1a). The striatum consists of several subregions including the NAc (Fig. 1b), each of which receives dopaminergic transmission from the midbrain and glutamatergic transmission from various areas of the cortex and the thalamus^11^. Furthermore, regional variations in the expression of the CB1 cannabinoid receptor have been reported within the rat striatum^12^. We therefore quantified the D2R immunofluorescence levels in each of the subregions of the striatum^12,13^. The expression levels of D2Rs were significantly increased in the NAc shell, core and ventral subregions of the striatum in *Disc1*-deficient mice compared to WT mice. In contrast, significant changes in D2R expression were not detected in the dorsal subregions of the striatum (Fig. 1c). We therefore focused on the D2Rs in the NAc for future experiments.

### Altered D2R supramolecular structure in the NAc of *Disc1*-deficient mice

**Figure 2:**
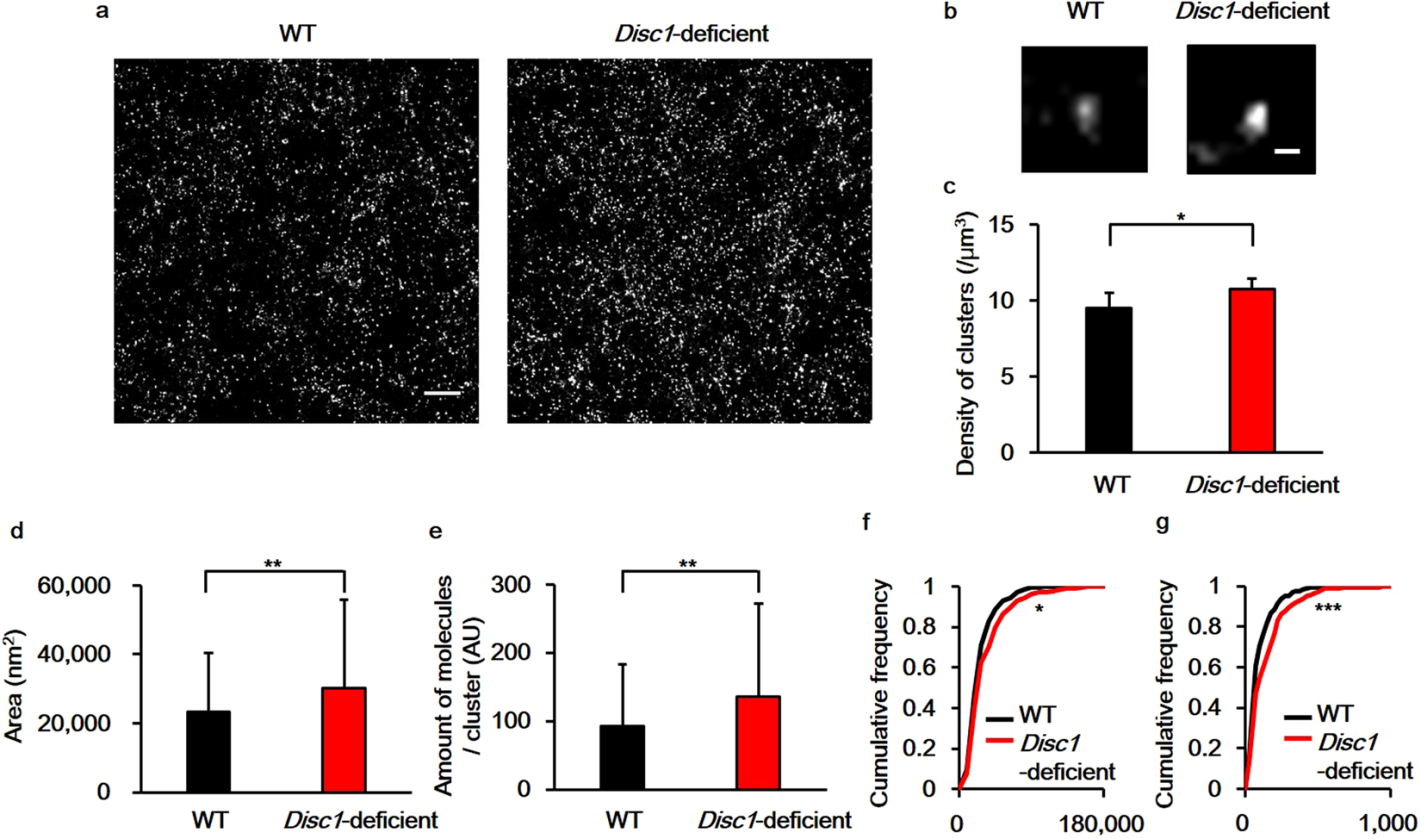
STORM imaging reveals changes in the spatial distribution of dopamine type 2 receptors (D2Rs) at the nanoscale in the striatum of *Disc1*-deficient mice. (**a**) Representative images of D2R nanostructures in the nucleus accumbens (NAc). Scale bar indicates 1.5 μm. (**b**) Higher resolution images of D2R nanoclusters in the NAc. Scale bar indicates 100 nm. (**c**) The density of D2R nanoclusters was quantified from 8 images from 4 mice for each genotype. Statistical significance was calculated using the Wilcoxon rank sum test (p = 0.015). Error bars indicate standard deviation (SD). (**d**) Quantification of the area of D2R nanoclusters. (**e**) Quantification of the number of D2R molecules in a nanocluster. A total of 240 clusters from 4 mice for each genotype were analysed for Fig. 2d and e, and the statistical significance was calculated using the Wilcoxon rank sum test (area: p = 0.0073, number of D2R molecules: p = 0.0012). Error bars indicate standard deviation (SD). (**f**) Cumulative frequencies of the area of nanoclusters. (**g**) Cumulative frequencies of the number of D2Rs in a nanocluster. A total of 240 clusters from 4 mice for each genotype were analysed for Fig. 2d and e, and the Kolmogorov–Smirnov test was used to test significance (area: p = 0.036, number of D2R molecules: p = 0.00063). *p < 0.05, **p < 0.01, ***p < 0.001.

We next studied the nanoscale spatial distribution of D2R molecules in the NAc using stochastic optical reconstruction microscopy (STORM), which has a lateral resolution of approximately 20 nm^9^. STORM images revealed that D2R molecules assemble to form nanosised clusters (nanoclusters) with an approximate diameter of several tens of nanometers (Fig. 2a and b). We compared spatial features of D2R nanoclusters in the NAc between WT and *Disc1*-deficient mice and found that the density of the D2R nanoclusters was approximately 1.1-fold higher in the NAc of *Disc1*-deficient mice compared to WT mice (Fig. 2c, WT: 9.51 ± 1.00/μm^3^; *Disc1*-deficient: 10.77 ± 0.72/μm^3^, n = 8 images for both genotypes, p = 0.015). In addition, the size of the D2R nanoclusters was approximately 1.3-fold larger in *Disc1*-deficient mice (Fig. 2d and f; WT: 0.0235 ± 0.0170 μm^2^; *Disc1*-deficient: 0.0302 ± 0.0257 μm^2^, n = 240 for both genotypes, p = 0.0073). Finally, the number of D2R molecules per nanocluster was approximately 1.3-times higher in *Disc1*-deficient mice (Fig. 2e and g; WT: 92.5 ± 90.6, *Disc1*-deficient: 135.4 ± 136.0, n = 240 for both genotypes, p = 0.0012).

### Clozapine’s effect on the supramolecular structure of D2R in *Disc1*-deficient mice

**Figure 3:**
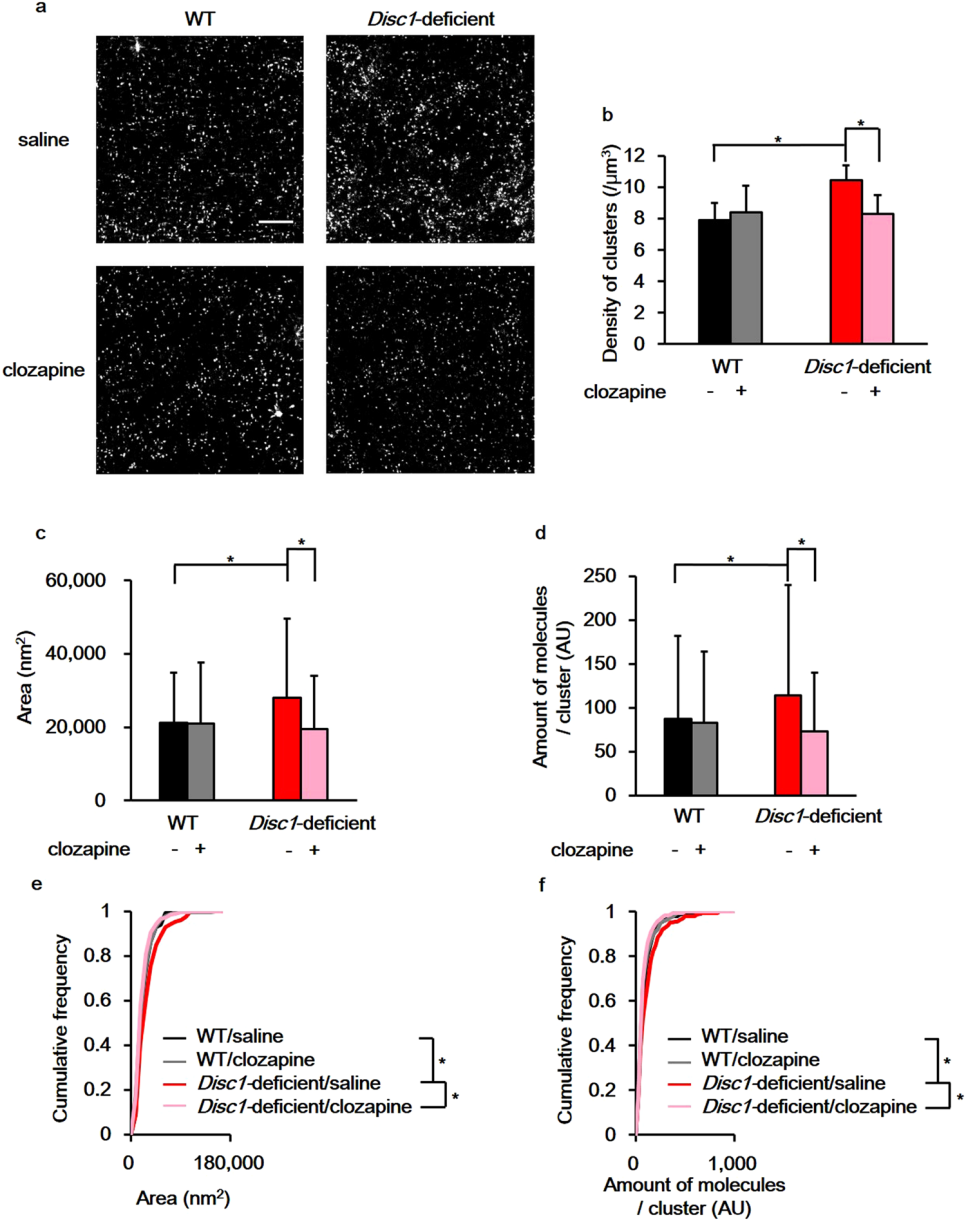
Clozapine reverses the changes in dopamine type 2 receptors (D2R) supramolecular assembly observed in the nucleus accumbens (NAc) of *Disc1*-deficient mice. (**a**) Representative images of D2R nanostructures in the NAc. Scale bar indicates 2.5 μm. (**b**) Quantification of D2R nanocluster densities. (**c**) Quantification of the D2R nanocluster area. (**d**) Quantification of the number of D2R molecules in a nanocluster. A total of 210 clusters from 3 mice for saline administrated groups and 180 clusters from 3 mice for clozapine administrated groups were analysed for Fig. 3b,c and d. + : clozapine administrated, −: saline administrated. Wilcoxon rank sum test with Benjamini-Hochberg procedure was used to test for significance (density: WT/saline–WT/clozapine; p = 0.29, WT/saline–*Disc1*-deficient/saline; p = 0.0012, WT/saline–*Disc1*-deficient/clozapine; p = 0.73, WT/clozapine–*Disc1*-deficient/saline; p = 0.022, WT/clozapine–*Disc1*-deficient/clozapine; p = 0.59, *Disc1*-deficient/saline–*Disc1*-deficient/clozapine; p = 0.0082, area: WT/saline–WT/clozapine; p = 0.32, WT/saline–*Disc1*-deficient/saline; p = 0.0031, WT/saline–*Disc1*-deficient/clozapine; p = 0.07, WT/clozapine–*Disc1*-deficient/saline; p = 0.00029, WT/clozapine–*Disc1*-deficient/clozapine; p = 0.47, *Disc1*-deficient/saline–*Disc1*-deficient/clozapine; p = 8.5 × 10^−16^, number of D2R molecules: WT/saline–WT/clozapine; p = 0.28, WT/saline–*Disc1*-deficient/saline; p = 0.0058, WT/saline–*Disc1*-deficient/clozapine; p = 0.73, WT/clozapine–*Disc1*-deficient/saline; p = 0.00018, WT/clozapine–*Disc1*-deficient/clozapine; p = 0.51, *Disc1*-deficient/saline–*Disc1*-deficient/clozapine; p = 0.0022). Error bars indicate standard deviation (SD). (**e**) Comparison of the cumulative frequency of the area of D2R nanoclusters. (**f**) Comparison of the cumulative frequency of the molecular expression of D2R in a nanocluster. To test for the significance, Kolmogorov–Smirnov test with Benjamini–Hochberg procedure was used (area: WT/saline–WT/clozapine; p = 0.37, WT/saline–*Disc1*-deficient/saline; p = 0.0059, WT/saline–*Disc1*-deficient/clozapine; p = 0.28, WT/clozapine–*Disc1*-deficient/saline; p = 0.0079, WT/clozapine–*Disc1*-deficient/clozapine; p = 0.48, *Disc1*-deficient/saline–*Disc1*-deficient/clozapine; p = 0.00011, number of D2R molecules: WT/saline–WT/clozapine; p = 0.46, WT/saline–*Disc1*-deficient/saline; p = 0.015, WT/saline–*Disc1*-deficient/clozapine; p = 0.25, WT/clozapine–*Disc1*-deficient/saline; p = 0.0097, WT/clozapine–*Disc1*-deficient/clozapine; p = 0.56, *Disc1*-deficient/saline–*Disc1*-deficient/clozapine; p = 0.00040). 210 clusters for saline administrated groups and 180 clusters for clozapine administrated groups were analysed for Fig. 3e and f. *p < 0.05.

We hypothesised that if the nanoscale alterations of D2R distribution underlie the molecular pathology of schizophrenia, these alterations could be reversed by administrating antipsychotics. Clozapine is an atypical antipsychotic that has been reported to improve psychiatric symptoms and cognitive function in patients with treatment-refractory schizophrenia^14^. To test whether clozapine administration would affect the localisation of D2Rs, spatial features of D2R nanoclusters in the NAc of *Disc1*-deficient mice were evaluated 2 weeks after clozapine treatment (Fig. 3a). Clozapine administration reduced the density of D2R nanoclusters in the NAc of *Disc1*-deficient mice (Fig. 3b; saline: 10.46 ± 0.93/μm^3^, n = 7; clozapine: 8.27 ± 1.21/μm^3^, n = 6, p = 0.0082), but had no effect on WT mice (Fig. 3b; saline: 7.91 ± 1.08/μm^3^, n = 7; clozapine: 8.39 ± 1.72/μm^3^, n = 6, p = 0.29). Furthermore, clozapine reversed the increased size of D2R nanoclusters (Fig. 3c and e; saline: 0.0280 ± 0.0216 μm^2^, n = 210; clozapine: 0.0195 ± 0.0145 μm^2^, n = 180, p = 8.5 × 10^−16^) and the number of D2R molecules per nanocluster in *Disc1*-deficient mice (Fig. 3d and f; saline: 114.1 ± 125.7, n = 210; clozapine: 72.7 ± 67.6, n = 180, p = 0.0022). In contrast, in WT mice, clozapine did not affect the size of nanoclusters (Fig. 3c and e, saline: 0.0212 ± 0.0136 μm^2^, n = 210; clozapine: 0.0210 ± 0.0166 μm^2^, n = 180, p = 0.32) or the number of D2R molecules per nanocluster (Fig. 3d and f; saline: 87.0 ± 94.9, n = 210; clozapine: 82.8 ± 81.5, n = 180, p = 0.283).

### Decreased spine numbers in the NAc of *Disc1*-deficient mice

Changes in the number and morphology of spines are indicated to be features of psychiatric disorders, and changes in spine properties have been extensively studied in cortical and hippocampal neurons. For example, changes in spine density have been found in the cortical neurons of schizophrenia patients^15^, and the density and morphology of spines are altered in hippocampal or cortical neurons in animal models of psychiatric disorders^16,17^. However, fewer studies have examined spines of medium spiny neurons (MSNs) in the NAc, and it is not established whether their properties are also affected in animal models of psychiatric disorders.

**Figure 4:**
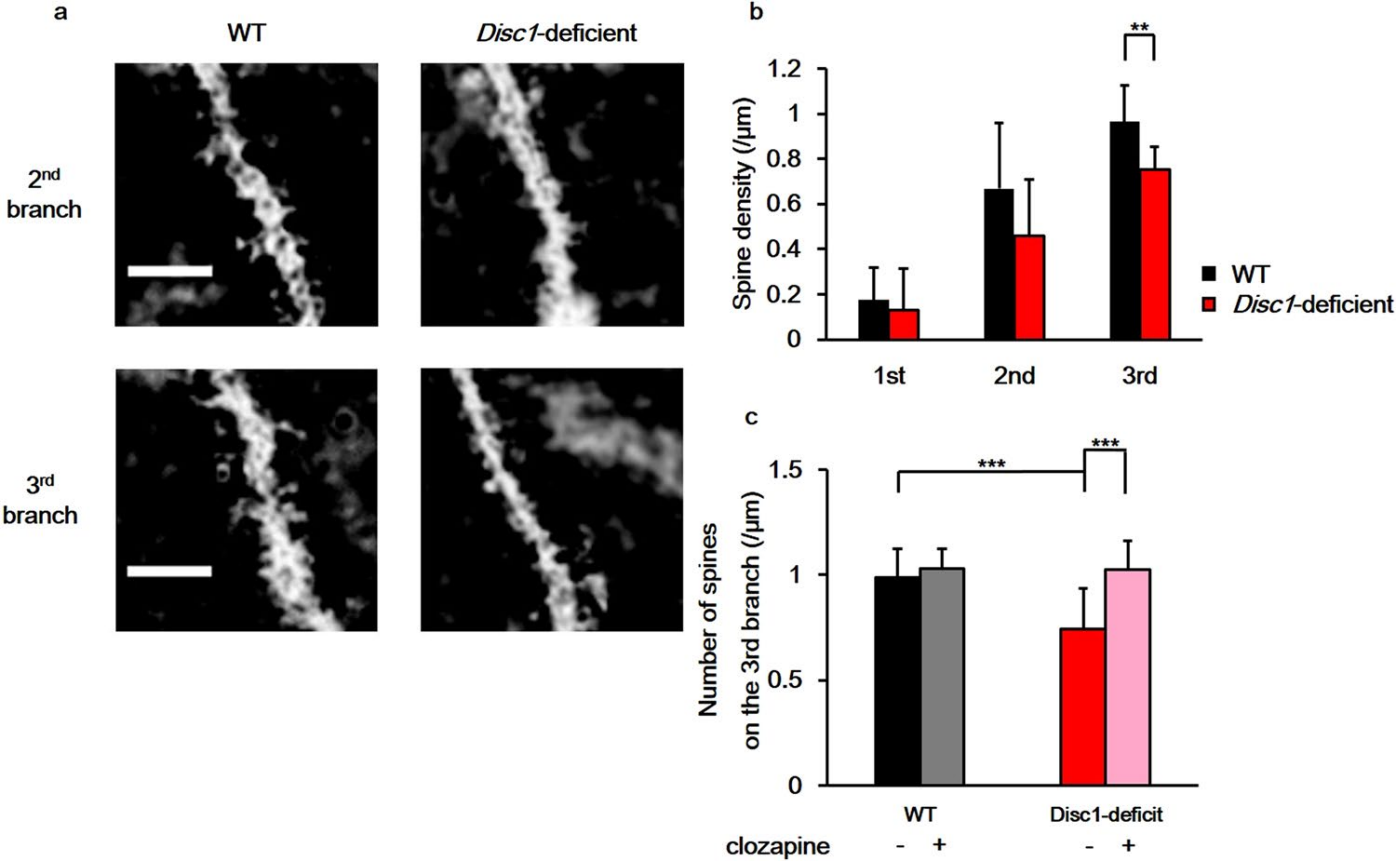
The number of dendritic spines of nucleus accumbens (NAc) medium spiny neurons (MSN) is reduced in *Disc1*-deficient mice. (**a**) Representative images of spines on the 2nd and the 3rd branches of MSN dendrites in the NAc. Scale bar indicates 4.0 μm. (**b**) Quantification of the number of dendritic spines on each branch of the MSN dendrites. A total of 12 dendrites from 2 mice for each genotype were counted and analysed using the two-tailed Student’s *t*-test (1^st^ branch: p = 0.530, 2^nd^ branch: p = 0.082, 3^rd^ branch: p = 0.0015). (**c**) The effect of clozapine on the number of spines on the 3rd dendrite of the NAc MSN. + : clozapine administrated, −: saline administrated. Spines on 12 dendrites from 2 mice for each group were counted and analysed using Tukey’s honestly significant difference post-hoc test (WT/saline–WT/clozapine; p = 0.90, WT/saline–*Disc1*-deficient/saline; p = 0.00070, WT/saline–*Disc1*-deficient/clozapine; p = 0.92, WT/clozapine–*Disc1*-deficient/saline; p = 0.000077, WT/clozapine–*Disc1*-deficient/clozapine; p = 0.99, *Disc1*-deficient/saline–*Disc1*-deficient/clozapine; p = 0.000092). Error bars indicate standard deviation (SD). **p < 0.01, ***p < 0.001.

We therefore investigated whether the spine density of MSN in the NAc was altered in *Disc1*-deficient mice using Golgi-Cox staining (Fig. 4a). We found that there were no differences in spine densities on the 1^st^ and 2^nd^ branches between the WT and *Disc1*-deficient mice (1^st^ branch, WT: 0.175 ± 0.140 spines/μm, *Disc1*-deficient: 0.131 ± 0.186 spines/μm, n = 12 dendrites from 12 neurons for both genotypes, p = 0.530; 2^nd^ branch, WT: 0.668 ± 0.289 spines/μm; *Disc1*-deficient: 0.459 ± 0.249 spines/μm, n = 12 dendrites from 12 neurons for both genotypes, p = 0.082). Interestingly, the spine density on the 3^rd^ branch of MSN dendrites was reduced by 22% in *Disc1*-deficient mice (Fig. 4b; WT 0.965 ± 0.159 spines/μm, *Disc1*-deficient 0.754 ± 0.101 spines/μm, n = 12 dendrites from 12 neurons for both genotypes, p = 0.0015).

We then asked whether the spine density on the 3^rd^ branch of dendrites would be restored after clozapine administration. The reduced spine density on the 3^rd^ branch of MSN dendrites in *Disc1*-mutant mice was indeed reversed by clozapine administration (Fig. 4c; saline: 0.744 ± 0.193 spines/μm, clozapine: 1.027 ± 0.132 spines/μm, n = 12 dendrites from 12 neurons, p = 0.000092). Similar to our results of D2R distribution, clozapine had no effect on MSN spine density in WT mice (Fig. 4c; 0.990 ± 0.136 spines/μm; clozapine: 1.030 ± 0.132 spines/μm, n = 12 dendrites from 12 neurons, p = 0. 90).

## Discussion

Using super-resolution microscopy, we found that *Disc1*-deficient mice had increased number of D2R nanoclusters, and that these D2R nanoclusters were larger and contained more D2R molecules than those in WT mice. Furthermore, these changes in D2R distribution were reversed after clozapine administration. Previous studies have shown that *Disc1*-deficient mice exhibited hypersensitivity to methamphetamine^6^ and that D2Rs played a central role in the acute sensitisation to methamphetamine^18^. Taken together, it is likely that the dopaminergic transmission through D2Rs in the NAc is enhanced in *Disc1*-deficient mice. In addition, the increased size and number of D2R nanoclusters may be indicative of enhanced dopaminergic transmission in the NAc of *Disc1*-deficient mice. Although the functional impact of the nanoscale distribution of D2Rs has not been directly tested, it has been shown that the nanoscale molecular distribution of receptor proteins affects synaptic transmission at glutamatergic synapses^8,10^.

In this study, we did not discern the location of D2Rs within neurons, thus, it would be premature to conclude that all the enlarged D2R nanoclusters contribute to the enhanced dopaminergic transmission. D2Rs localised in various parts of neurons are known to execute different functions. For example, different isoforms of D2Rs are expressed at pre- and postsynaptic sites (D2S and D2L, respectively)^19^. The D2R antibodies used in the present study cannot distinguish between these isoforms. Future development of isomer-specific antibodies would clarify which D2R isomers are present in the nanoclusters and are affected in psychiatric disorders. We should note that the D2Rs observed in this study may include ones expressed both on the cell surface as well as within the cell. Although D2Rs within the cell would not be involved in neurotransmitter signalling, a recent study revealed that endocytosed D2Rs can contribute to the regulation of spine formation by activating signalling pathways such as ERK signalling^20^.

It has been recently shown that DISC1 modulates the D2R signaling. Therefore the alteration of the nanoscale structures of D2R in DISC1 deletion mice might be due to loss of this modulation of D2R signaling. D2Rs activate G-protein dependent and non-G protein signaling pathways. The latter is elicited when D2Rs form a complex with β-arrestin, which leads to the internalisation of D2R through clathrin-dependent endocytosis and the activation of glycogen synthase kinase 3^21–23^. In striatal neurons, DISC1 binds to the D2R/β-arrestin complex and inhibits D2R internalisation^24^. When this D2R–DISC1 interaction is inhibited, the recruitment of clathrin to the D2R/β-arrestin complex is enhanced and D2R internalisation is increased^24^. This modulation of D2R internalisation by DISC1 could affect D2R molecular dynamics, resulting in structural changes of the nanoclusters. We postulate that the increase in the size and density of the D2R nanoclusters in *Disc1*-deficient mice could be due to the loss of DISC1-mediated inhibition of D2R internalisation. Moreover, it has been reported that clozapine inhibits the formation of the D2R/β-arrestin complex^25^. Interestingly, we found that clozapine reverses the changes in D2R nanostructure observed in *Disc1*-deficient mice, which further supports the hypothesis that D2R/β-arrestin complex-dependent signalling pathways are involved in the regulation of D2R nanoclusters.

Although the role of MSN spine density in the NAc in psychiatric disorders has received less attention than cortical and hippocampal neurons^15,16^, our results suggest that the spine density of NAc MSN may be an endophenotype of psychosis. The restoration of reduced spine density in *Disc1*-deficient mice caused by clozapine treatment suggests that neural circuits of the NAc could be therapeutic targets of this drug. This is consistent with the results of a recent study that examined DISC1-Q31L mutant mice; these mutant mice showed reduced spine density in MSN, which was partially recovered by administration of D2R antagonists^26^. While regulation of spines via D2R activation has not been directly shown, the above results suggest that D2R activity may indeed influence the spine density of MSN. Furthermore, because D2R activation in MSN is indispensable for long-term depression (LTD) induction^27^, and changes in spine morphology are often accompanied by long-term plasticity (both long-term potentiation and LTD in hippocampal neurons), we hypothesise that D2Rs regulate spine morphology in MSN^28^.

Although this study did not directly determine the causative roles of D2R nanoclusters in expression of psychiatric symptoms, it is quite possible that the clustering of D2Rs contributes to the symptoms through some mechanisms. One possible mechanism is altered dopaminergic transmission. As discussed above, the enhanced D2R clustering may change synaptic transmission and plasticity by enhanced D2R signalling, leading to abnormal behaviour. Another mechanism might be cytotoxicity of D2R clusters as is commonly supposed for pathological roles of protein aggregation in neurodegenerative disorders. However, this possibility seems unlikely because there was no indication of neurodegeneration in the striatum slice preparation of *Disc1*-deficient mice. In addition, even the normal mice have moderate amounts of D2R nanoclusters, suggesting that a D2R nanocluster *per se* is not cytotoxic.

In this study, we found novel morphological changes in D2R assembly in a mouse model of psychiatric disorders. As a measurable and quantifiable index of the disease state, the alteration of D2R nanoclusters might be useful for diagnosing psychiatric disorders. In addition, as we have shown for clozapine, the alteration of D2R nanoclusters might be used as an index in the screening and evaluation of drugs for psychiatric disorders. Furthermore, because it is predicted that alterations of nanoscale molecular distributions will be observed in models and patients of other disorders, nanostructures formed by molecules could be indicators of pathological phenotypes of disorders. Indeed, another super-resolution technique, expansion microscopy, has enabled precise pathological diagnosis^29^. The new pathology focusing on nanoscale molecular distribution might be a powerful tool for the clinical application.

## Methods

### Animals

Animal experiments were carried out in accordance with the institutional regulations for animal experiments based on the governmental Guideline for Proper Conduct of Animal Experiment and Related Activities, and were approved by the institutional Committee of the University of Tokyo. *Disc1*-deficient mice^6^ were provided by the RIKEN BioResource Center (Tsukuba, Japan) through the National Bioresource project of the MEXT, Japan. Mice had free access to food and water. Male and female mice aged 12–15 weeks were used.

### Immunohistochemistry

Mice were deeply anaesthetised with 0.1 mL Somnopentyl (Kyoritsu Seiyaku, Tokyo, Japan) and transcardially perfused with 4% paraformaldehyde (PFA) in 0.1 M phosphate buffer saline (PBS) containing 137 mM NaCl, 2.68 mM KCl, 0.775 mM Na_2_HPO_4_ and 1.47 mM KH_2_PO_4_, pH 7.4. The brains were removed and post-fixed in 4% PFA in PBS overnight at 4 °C, then cryoprotected with 30% sucrose for 3 days at 4 °C. Brains were frozen in Tissue Freezing Medium (Leica Biosystems Nussloch GmbH, Nußloch, Germany) and stored at −80 °C until use. 10 µm thick sections were cut on a cryostat (CM1900; Leica Microsystems GmbH, Wetzlar, Germany), collected on either Matsunami aminosilane (MAS) coated glass slides (S9215; Matsunami Glass Ind, Osaka, Japan) for conventional fluorescence microscopy, or MAS coated coverslips (thickness < 0.12 mm; Matsunami Glass Ind, Osaka, Japan) for STORM imaging, and then stored at −20 °C. The striatum was identified according to the mouse brain atlas^15^.

Brain sections were incubated in an antigen retrieval solution containing 0.03% pepsin (Agilent Technologies, Santa Clara, CA), 0.3% Triton X-100 (Nacalai Tesque, Kyoto, Japan), and 0.2 M HCl for 10 min at room temperature, and then in blocking buffer containing 3% bovine serum albumin (Nacalai Tesque) and 0.3% Triton X-100 in PBS for 20 min at room temperature with constant gentle shaking. Brain preparations were incubated with rabbit anti-D2R antibody diluted in blocking buffer (1:50 in blocking buffer; Frontier Institute, Ishikari, Japan) overnight at 4 °C. The following day, sections were washed and incubated with goat anti-rabbit IgG secondary antibody (1:500 or 1000 in blocking buffer; Jackson Immunoresearch, West Grove, PA) conjugated to Alexa647 (Thermo Fisher scientific, Waltham, MA) for 1 h at room temperature with constant gentle agitation. Brain sections used for STORM imaging were then post-fixed with 4% PFA in PBS for 20 min at room temperature and washed with PBS. For fluorescence microscopy, labelled brain sections were mounted in Fluoro-KEEPER Antifade Reagent with DAPI (Nacalai Tesque), coverslipped, and sealed with nail polish.

### Fluorescence microscopy

Fluorescent images were acquired using an Olympus IX71 inverted microscope (Olympus, Tokyo, Japan) with a 4× objective (UPlan FI 4×/0.13; Olympus), an iXon EMCCD camera (Andor, Belfast, United Kingdom), and a fluorescent filter set (Cy5-4040C; Semrock, Lake Forest, IL).

### STORM imaging

After immunostaining, 200 nm-fluorescent beads (F-8810; Thermo Fisher Scientific) were placed on the brain sections as spatial fiducial markers. The beads were prepared by diluting the beads suspension 100-fold with PBS, then by another 50-fold with PBS containing 50 mM MgCl_2_. Sections were incubated with the beads for 20 min at room temperature in the dark. Immediately before STORM imaging, brain sections were mounted in a solution containing 50 mM HEPES (pH 8.0), 10 mM NaCl, 10% glucose, 60% sucrose, 1% 2-mercaptoethanol, 0.5 mg/mL glucose oxidase and 0.04 mg/mL catalase, coverslipped and sealed with nail polish. A series of 64,000 images was acquired at 62.5 Hz and illuminated with OBIS 6.6 kW/cm^2^ 640 nm laser (Coherent, Santa Clara, CA). STORM imaging was performed on a custom-made direct STORM microscope^8^ equipped with an UPlanSApo NA1.40 100× objective lens (Olympus) and an iXon 860 EMCCD camera (Andor). After taking 10,000 frames, OBIS 405 nm laser beam (Coherent) was irradiated to re-activate the Alexa647 fluorophore with 1 W/cm^2^. The intensity of the 405 nm laser beam was doubled every 10,000 frames until it reached 32 kW/cm^2^. The 64,000 image series was analysed to determine the localisation of fluorophores using ImageJ software (NIH, Rockville, MD) and a custom plugin. Based on single-molecule localisation datasets, STORM images were reconstructed using 30 nm binning.

### Clozapine administration

Stock solution of clozapine was prepared by dissolving of 20.8 mg clozapine (Nacalai Tesque) in 1.24 mL of 0.1 M HCl, and pH was adjusted to approximately 7.0 by adding 0.61 mL of 0.1 M NaOH. The solution was then diluted to 0.1 mg/mL clozapine with 0.9% saline. Mice were intraperitoneally injected with clozapine (5 mg/kg) or saline every day for 2 weeks. Mice were sacrificed one day after the last clozapine or saline administration.

### Golgi-Cox staining

The procedure of Golgi-Cox staining was followed as previously described^30^. To prepare brains for the staining, mice were deeply anaesthetised with 0.1 mL Somnopentyl (Kyoritsu Seiyaku) and transcardially perfused with PBS. Brains were removed, washed with PBS, and incubated away from light in 20 mL of Golgi-Cox solution containing 5% K_2_Cr_2_O_7_, 5% K_2_CrO_4_ and 5% HgCl_2_ in pure water, for 24 h at room temperature. After the initial 24 h immersion, brains were moved into fresh Golgi-Cox solution and further incubated at room temperature for 10 days in the dark. The brains were then transferred to a tissue-protectant solution containing 0.276% NaH_2_PO_4_, 2.76% Na_2_HPO_4_, 1.8% NaCl and 0.54 M NaOH in pure water, pH 7.2 and stored at 4 °C in the dark. After 24 h, brains were moved into a fresh tissue-protectant solution and incubated for 7 days at 4 °C in the dark. Finally, the brains were cut into 150 μm-thick sections on a PRO 7 vibratome (Dosaka EM, Kyoto, Japan), immersed in tissue-protectant solution and stored at 4 °C in the dark for 3 days.

Staining stepswere performed in the dark as previously described^30^. First, the sections were rinsed twice with pure water for 5 min each, and then immersed in 50% ethanol for 5 min. Second, the sections were incubated in 67% NH_3_ solution (prepared by mixing 30 ml NH_3_ solution (Nacalai Tesque) with 15 mL pure water) for 8 min at room temperature, and then rinsed twice with pure water each time for 5 min. Third, the sections were incubated in 5% Na_2_SO_3_ solution for 10 min at room temperature followed by 1 min washes. Finally, the sections were sequentially dehydrated in 50%, 95% and 100% ethanol for 6 min each and washed in xylene for another 6 min. Sections were mounted in Malinol (Muto Pure Chemicals, Tokyo, Japan), coverslipped, and sealed with nail polish. Specimens were dried for at least 1 h at room temperature before imaging.

### Data analysis

Changes in D2R expression in the striatal subregions were analysed using the two-tailed Student’s *t*-test. Differences in the density, area and the expression of D2R clusters between WT mice and *Disc1*-deficient mice were analysed using the Wilcoxon rank sum test. Kolmogorov–Smirnov test was utilised to compare the cumulative frequency of the area and the expression of D2R clusters. The differences in the densities, the area and the expression of D2R clusters between WT mice and *Disc1*-deficient mice after clozapine administration was analysed using Wilcoxon rank sum test with Benjamini-Hochberg procedure^31^. Kolmogorov–Smirnov test with Benjamini-Hochberg procedure was adopted to compare the cumulative frequency of the area and expression of D2R clusters. Changes in spine density were analysed using the two-tailed Student’s *t*-test, Kruskal–Wallis rank sum test and Tukey’s HSD post-hoc test. Data analysis was performed in the R environment (the R foundation) and in Excel (Microsoft, Redmond, WA).The datasets generated or analyzed during the current study are available from the corresponding author on reasonable request.
